# Supplementary material for: Self-stigmatization and treatment preferences: Measuring the impact of treatment labels on choices for depression medications
Source: PLoS One. 2024 Sep 3;19(9):e0309562. doi: 10.1371/journal.pone.0309562 (PMC11371228; doi:10.1371/journal.pone.0309562)
Supplement: S1 Appendix — (DOCX) [file pone.0309562.s001.docx]

About you

B1. What year were you born? (Enter as YYYY) [Allowable response range 90-18. Terminate if above 80 or less than 18]

B2. Has your doctor or any other health care provider told you that you have any of the following?

- Alzheimer’s disease or dementia
- Anemia
- Heart murmur
- Depression
- Epilepsy
- Multiple Sclerosis
- Atopic dermatitis (Eczema)
- Prostate Cancer
- Skin Cancer
- None of the above

[If B2=Depression, Continue. Otherwise, Terminate.]

B3. When were you first diagnosed with depression?

- Less than 2 years ago
- More than 2 years, but less than 5 years ago
- More than 5 years but less than 10 years ago
- More than 10 years but less than 15 years ago
- More than 15 years ago

B4. Has your doctor prescribed more than one oral medicine at a time because your depression symptoms were not improving as expected?

- Yes
- No [Terminate]
- Don’t know or not sure [Terminate]

Informed Consent

**Consent**

Thank you for your interest in this health survey. The goal of this survey is to quantify how treatment information affects patients stated treatment preferences. The survey is being conducted by the XXXXXX.

In this survey, you will be asked questions about your experience with depression. You will be asked to read about different features of treatment for depression, and then asked to respond to a series of choice questions about your willingness to accept therapies based on varying product-label information. This survey will take about 20 minutes for you to complete.

The potential risks from completing this survey are minimal. There are no physical risks associated with taking the online survey but there is, however, the potential loss of confidentiality. Every effort will be made to keep your information confidential and even if your

information is accessed, no one can link it to you personally. You have the right to skip any questions you don’t want to answer. There is no direct benefit from completing the survey. The reason you might want to participate is to help doctors and government officials make better decisions to help people with depression.

Participation in this survey is completely voluntary. You can decide to take part or not. If you decide to take part, you can change your mind and stop at any time. Your careful consideration of each question in this survey is very important for the success of this study.

For questions about the study or if you have problems, concerns, questions or suggestions about the research, contact the study PI, XXXX. For questions about your rights as a research participant, or to discuss problems, concerns or suggestions related to the research, or to obtain information or offer input about the research, you may also contact the XXXX Review Board (IRB) Office at (XXX) XXX-XXXX.

Select one answer only:

- I agree to participate in this study [This button takes respondent to the survey]
- I do not agree to participate in this study [Next question]

Your opinions are very important to us. Are you sure that you don’t want to participate in this study?

- I agree to participate in this study [This button takes respondent to the survey]
- I do not agree to participate in this study [Show respondents termination page]

About You

B5. What have you done to help manage your depression? *(Check all that apply.)*

- Taken oral (by mouth) medicines prescribed to me by a physician
- Received injectable medicines
- Talked with a psychologist or therapist
- Acupuncture
- Meditation
- Herbal medicine or dietary supplements
- Changed my diet
- Electromagnetic therapy
- Other

[If B5 = Oral medicines or Injectable medicines]

B6. Are you currently taking medicines to manage your depression?

- Yes
- No
- Don’t know or not sure

B7. Would you say you are currently experiencing depression symptoms?

- Yes
- No
- Don’t know or not sure

B8. What is your current weight?

_____ pounds [80-500 lbs. allowable range and soft validation prompt (give them a chance to fix if outside, otherwise terminate.]

B9. How tall are you? feet inches [3-7 feet allowable range.]

In this survey we will ask you to think about treatments that doctors might suggest for patients whose depression has not improved enough after taking antidepressants for several months.

In the next few pages, we will tell you about the effects that these medicines could have on depression and a patient’s health.

**Severity of Depression Symptoms**

Different people have different experiences with depression. Later in this survey we will ask you to think about treatments for the worst depression symptoms you have ever experienced.

1. Which of these descriptions most closely describes the worst depression symptoms you have ever experienced?

| **Severity** | **Symptom Description** |
| --- | --- |
| - Very Severe | All the time:   - feel very sad, miserable and hopeless - feel dread and anguish, often with an overwhelming sense of panic - Unable to feel any anger, grief, pleasure, or other emotions even for relatives and friends - have unshakable beliefs of remorse, guilt, and worthlessness |
| - Severe | Most of the time   - feel sad and gloomy, but sometimes brighten up - feel tense with occasional panic, but you can overcome these feelings with some difficulty - feel a lack of interest in your surroundings, friends, and acquaintances - feel guilty, inferior and pessimistic about the future |
| - Moderate | Occasionally:   - feel sad or low, but brighten up without difficulty - feel on edge, tense or uneasy for no clear reason - feel a limited ability to enjoy things that would usually interest you - feel guilty, self-critical, or consider yourself a failure |

1. Which description above most closely describes your depression symptoms over the past 2 weeks?
   - Very severe
   - Severe
   - Moderate
   - None of the above

**Medicines for Depression**

# Medicine Feature: How often the medicine has worked well

In this survey we will ask you to think about medicines that doctors might suggest for patients whose treatment has not worked well after taking antidepressants for several months.

In this survey “worked well” means

- - - having a normal interest in your surroundings and other people
    - generally feeling calm
    - having few pessimistic thoughts
    - feeling sad only in particular circumstances

Different people can respond differently to a treatment. Therefore, doctors do not know if a treatment will work well for a particular person. However, based on their experience treating large groups of people, doctors know how well a medicine worked for people like you.

2a. Have you ever had a treatment for your depression that worked well, at least for some period of time?


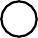
 Yes


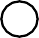
No


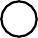
Don’t know or not sure

We will use pictures to help you think about how well a treatment has worked for people like you. The picture below has 100 figures. Each figure is a person treated for depression. The **orange** figures show for how many people a medicine worked well.


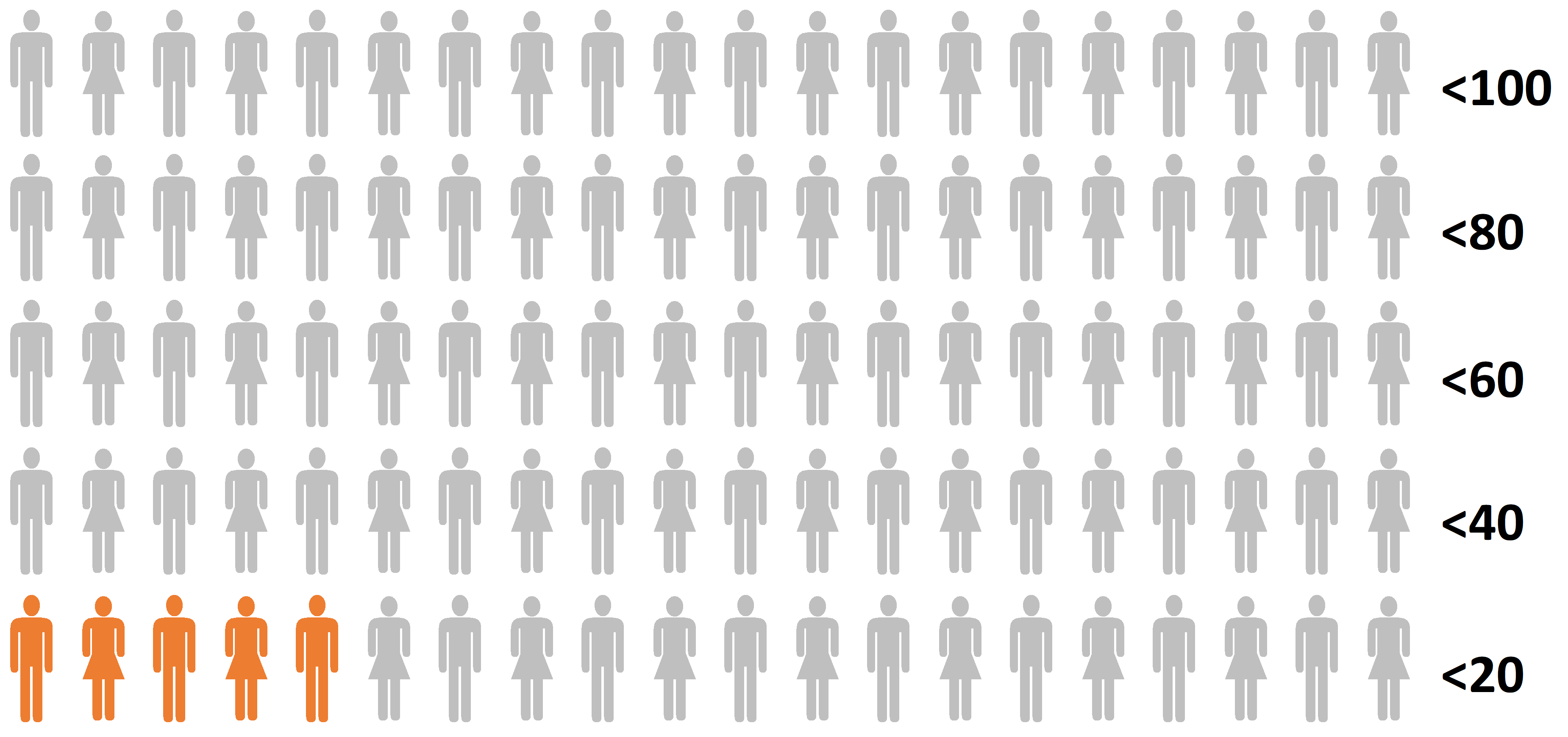


- 5 figures are **orange**. That means the medicine worked well for 5 out of 100 (5%) of people who got it.
- 95 figures are **gray**. That means the medicine did not work well for 95 out of 100 (95%) of people who got it.

When there are more orange figures, there is a greater chance the medicine would work well for other people who get it.

Please use this picture to answer the question below.


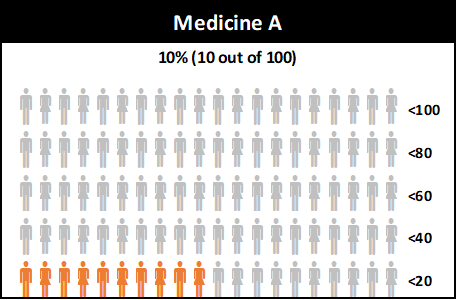


1. For how many people did the medicine work well?
   - 20 people out of 100 (20%)
   - 90 people out of 100 (90%)
   - 10 people out of 100 (10%)
   - 5 people out of 100 (5%)

[If Q3!=10 people out of 100 (10%)]


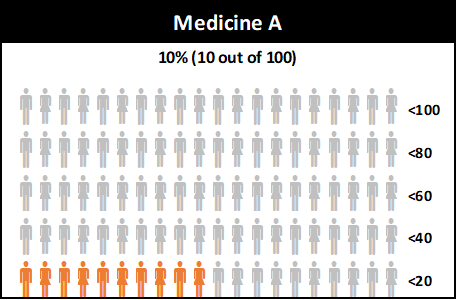
**Let’s look at that question again**

Remember that the figures in orange represent people for whom the medicine worked well. There are for 10 figures in orange in the picture above. That means Medicine A worked well for 10 out of 100 (10%) of people who got it.

**Medicine Information Contained in Package lnserts**

In this survey, we will ask you to think about medicines for depression based on the kind of information contained in the package insert patients get with their prescription medicines.

The insert includes details on the medicine that are considered important to understand how a medicine works, how it is taken, and possible health problems that have resulted from taking the medicine.

Here is a picture of how the information is presented in an insert.


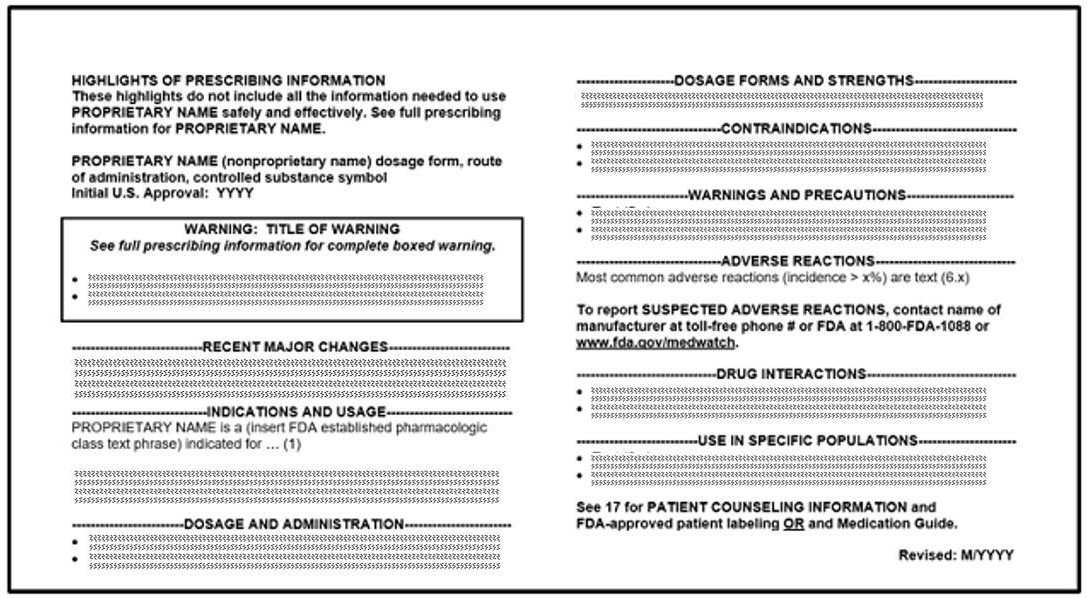


# Insert Information: Medicine type

The first part of the insert often describes the type of medicine. Medicines of a particular type work in a similar way.

Your doctor could recommend adding another medicine if your symptoms do not improve enough with your current antidepressant medicine. Possible options could include a different antidepressant, or another type of medicine such as an **atypical antipsychotic** or a **serotonin-dopamine activity modulator**.

1. Has a doctor ever suggested giving you an anti-psychotic medicine for your depression or some other condition, such as sleeplessness?
   - Yes
   - No
   - Don’t know or not sure
2. Has a doctor ever suggested giving you a serotonin-dopamine activity modulator for your depression or some other condition?


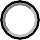
 Yes
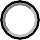
 No


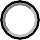
 Don't know or not sure

[Randomly assign this page to respondents or not. Maintain balanced assignment ~50%/50%]

A patient does not need to have schizophrenia with psychotic episodes (for example, seeing things that are not there or hearing voices) to use an atypical antipsychotic. Atypical antipsychotics have been approved to treat depression because they can be effective in reducing symptoms and avoid some of the side effects found in older antipsychotics.

Adding an atypical antipsychotic or serotonin-dopamine modulator can help people with depression symptoms that do not improve enough with a single antidepressant.

# Medicine Feature: Approved uses

Next, the insert includes a list of health problems the government has approved the medicine for. Some medicines that have been approved for treating major depressive disorder also have been approved for treating other conditions.

Some of the medicines we will ask you to think about in this survey have been approved to treat other health problems in addition to major depressive disorder.

Here is an example from a medicine insert. Under the section “Indications and Usage” the insert includes information about the type of medicine class and the health problems or indications this medicine has been approved for use.


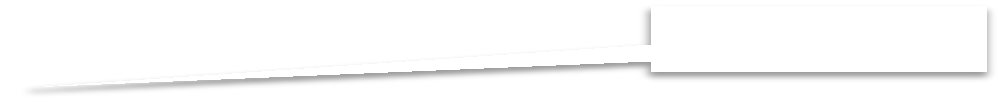

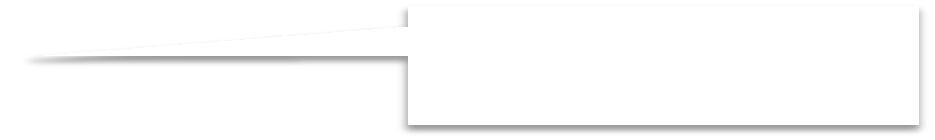


Type of medicine

**----------------------------INDICATIONS AND USAGE------------------------------**

This treatment is an ***atypical antipsychotic*** indicated for the treatment of:

- Major depressive disorder
- Schizophrenia

Approved health problems it

can be used to treat

1. What type of treatment is this medicine?
   - Antidepressant
   - Atypical antipsychotic
   - Serotonin-dopamine activity modulator
   - Don’t know or not sure
2. Which of the following health problems is this medicine approved to treat?

(*Check all that apply.)*

- - Major depressive disorder
  - Anxiety disorder
  - Post-traumatic stress disorder
  - Attention-deficit/hyperactivity disorder
  - Schizophrenia
  - Bipolar disorder
  - Dementia

[If Q6!=Atypical antipsychotic or Q7!=Major depressive disorder and Schizophrenia]

**Remember**

The medicine insert shows the type of treatment and a list of indications for the medicine.


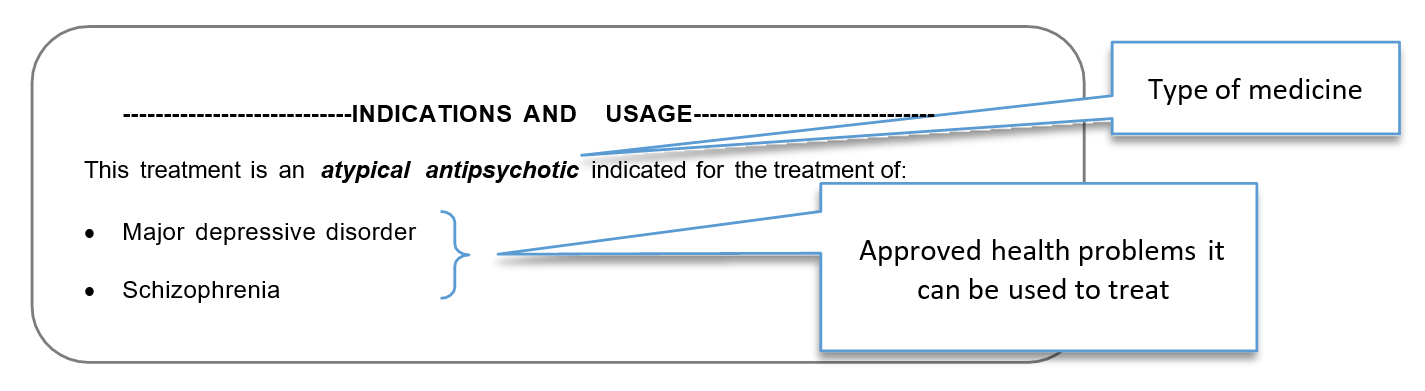


In the picture above, the insert shows that this is an atypical antipsychotic. It also shows that the medicine is approved for the treatment of major depressive disorder and schizophrenia.

# Thinking about taking an additional medicine

Suppose that you have been depressed for two months with the worst depression symptoms you have ever experienced. During that time, you had taken an antidepressant to help improve your mood, but the medicine did not work well to improve your mood even though you took the medicine correctly. Your doctor then prescribed a different antidepressant that also did not work well.

Suppose your doctor suggests two new alternative medicines that you would take in addition to your current medicine (Medicine A or Medicine B).


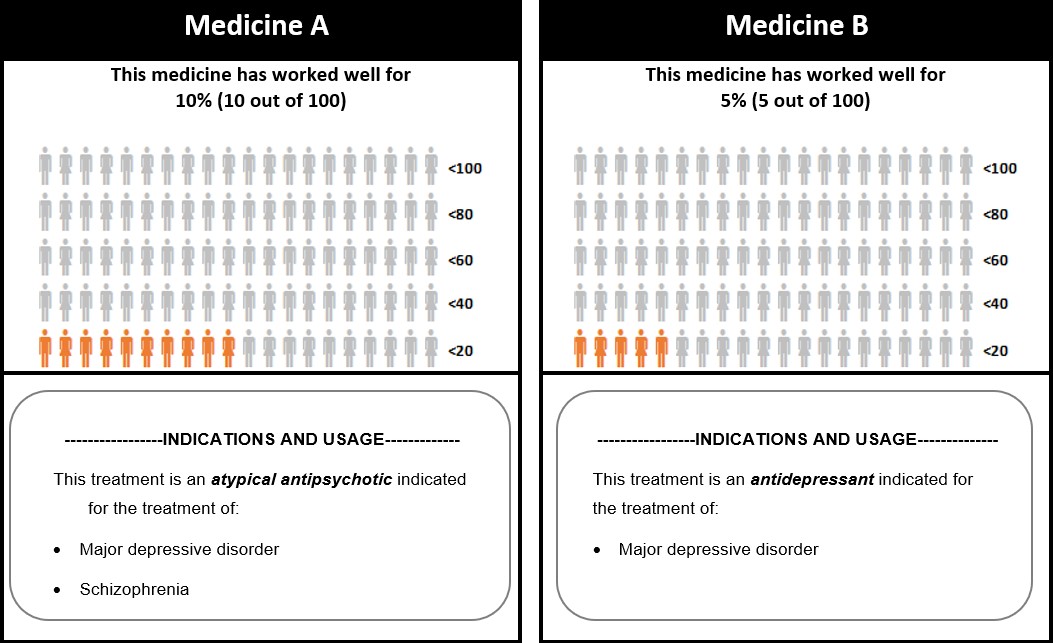


1. Which medicine has worked better to improve people’s mood?
   - Medicine A
   - Medicine B
   - Both Medicine A and Medicine B have worked about the same
   - Don’t know or not sure
2. Which medicine has been approved to treat more than one kind of health problem?
   - Medicine A
   - Medicine B
   - Both Medicine A and Medicine B have worked about the same
   - Don’t know or not sure

[If Q8!=Medicine A or Q9!=Medicine A]

**Let’s look at that question again**


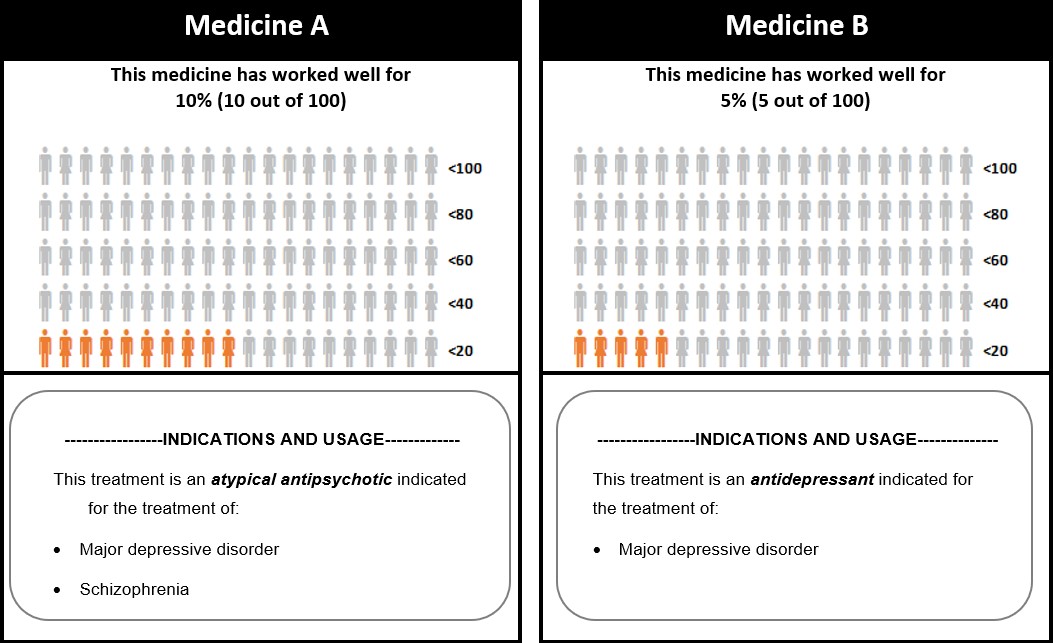


Remember that the figures in orange represent people for whom the medicine worked well. In the picture above, there are for 10 figures in orange for Medicine A, and 5 figures in orange for Medicine B. That means Medicine A worked well for 10 out of 100 (10%) of people who got it, while Medicine B worked well for 5 out of 100 (5%) of people who got it. Medicine A worked well for more people.

Also, the insert for Medicine A states that it is approved to be used to treat major depressive disorder and schizophrenia. Medicine B is only approved for the treatment of major depressive disorders.

**
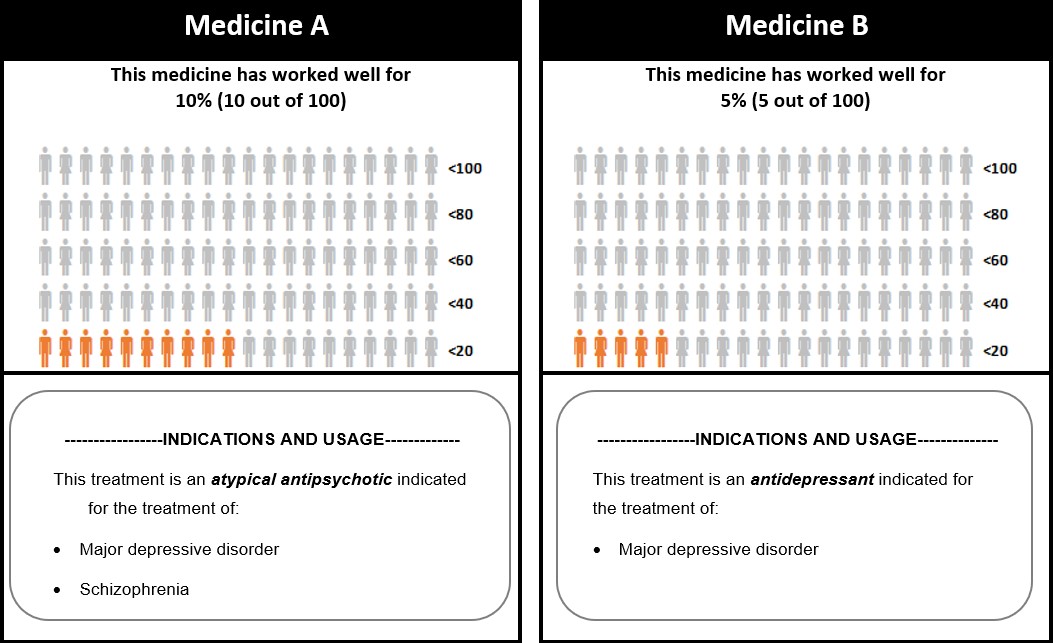
Again, consider the same options**

1. Which of these medicines would you try **first** if these were the only alternatives?
   - Medicine A
   - Medicine B
   - Don’t know or not sure

# Medicine Feature: Dosage and administration

Another section of the insert shows how people should take the medicine.

Some of the medicines we will show you later in the survey are taken as a pill that you take with water at home once a day. Other medicines are taken as an injection or shot in your arm once a month at your doctor's office or clinic.

Again, suppose your doctor suggests two new alternative medicines that you could take in addition to a current antidepressant (Medicine A or Medicine B).


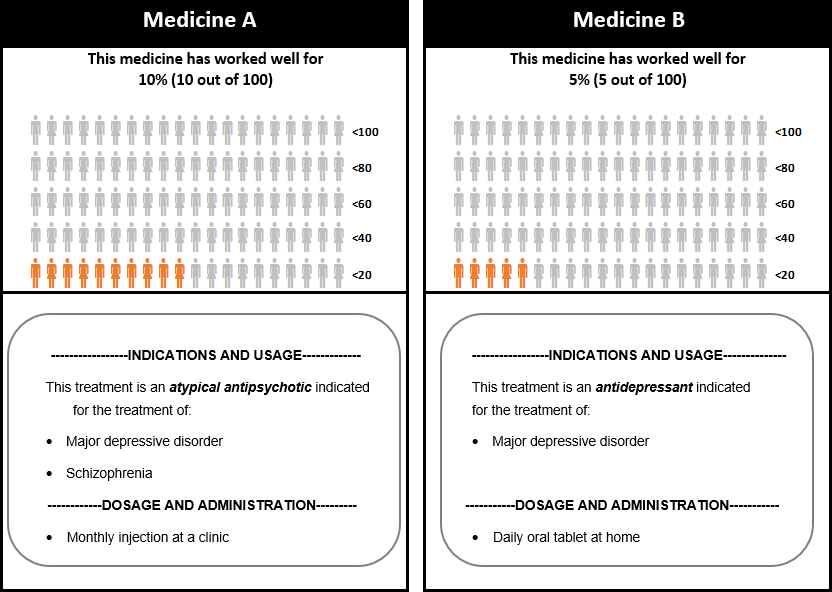


1. Which of these medicines would you try **first** if these were the only alternatives?
   - Medicine A
   - Medicine B
   - Don’t know or not sure

# Medicine Feature: Warnings and precautions

The insert also will include a section highlighting the potential side effects from medicines to treat depression.

Some people who have taken medicines to treat depression gained weight within a year of starting the medicine. These changes in weight occurred without any

changes in the person’s diet or lifestyle. For the medicines you will see in this survey, we have calculated how much weight you would gain based on the weight you provided before.

Other people who have taken medicines to treat depression had a side effect called akathisia
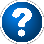
.

**[if respondent clicks on question mark in previous page]**

Akathisia is a movement disorder that makes it hard for you to stay still. It causes an urge to move that you can't control. If you have akathisia, you need to fidget all the time, walk in place, or cross and uncross your legs. This side effect would last as long as you take the medicine.

1. Have you ever had akathisia as a medicine side effect?


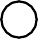
 Yes
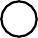
 No


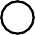
 Don't know or not sure

Below is an example insert showing warnings and precautions:

**-------------------INDICATIONS AND USAGE-------------------**

This treatment is an **atypical antipsychotic** indicated for the treatment of:

- Major depressive disorder
- Schizophrenia

**-------------DOSAGE AND ADMINISTRATION------------**

Monthly injection at a clinic

**------------WARNINGS AND PRECAUTIONS-------------**

- Most patients have experienced a 2% increase in weight ([Enter calculated weight (B7*0.02)] lbs.)
- Most patients have experienced akathisia

1. How much weight have people gained within 1 year when they took Medicine A to treat depression symptoms?

- 2% or [Enter calculated weight (B8*0.02)] lbs.
- 5% or [Enter calculated weight (B8*0.05)] lbs.
- 7% or [Enter calculated weight (B8*0.07)] lbs.
- They did not gain any weight
- Don’t know or not sure

**Your Preferences for Depression Medicines**

In the next part of this survey, we will ask you **X** [Enter number of questions in the experimental design file] questions in which you will compare Medicine A and Medicine B.

As you consider your choices, please keep these things in mind:

- Remember we asked you to assume that you have been very depressed recently and your symptoms have not improved enough with your current medicine.
- Assume your doctor is making a recommendation of adding another medicine to help with your symptoms. The medicines we will show you would be taken in addition to a regular antidepressant.
- Assume that all medical bills, including the cost of the medicines, are covered by health insurance.
- The medicines we will show you do not necessarily describe medicines that currently exist. However, such medicines could be developed in the future.

We understand that your own experiences with medicines for depression are complicated. Please think carefully about these medicines even if your own experience with depression medicines is different from the medicines that we describe. In these questions, please assume that the medicine effect will be as described, not better or worse.

**Your answers are very important for this study. They will help doctors make better decisions when caring for people living with depression.**

[Example choice question. Enter questions based on experimental design provided separately]

Suppose your doctor suggests two new alternative medicines that you could take in addition to a current antidepressant (Medicine A or Medicine B).


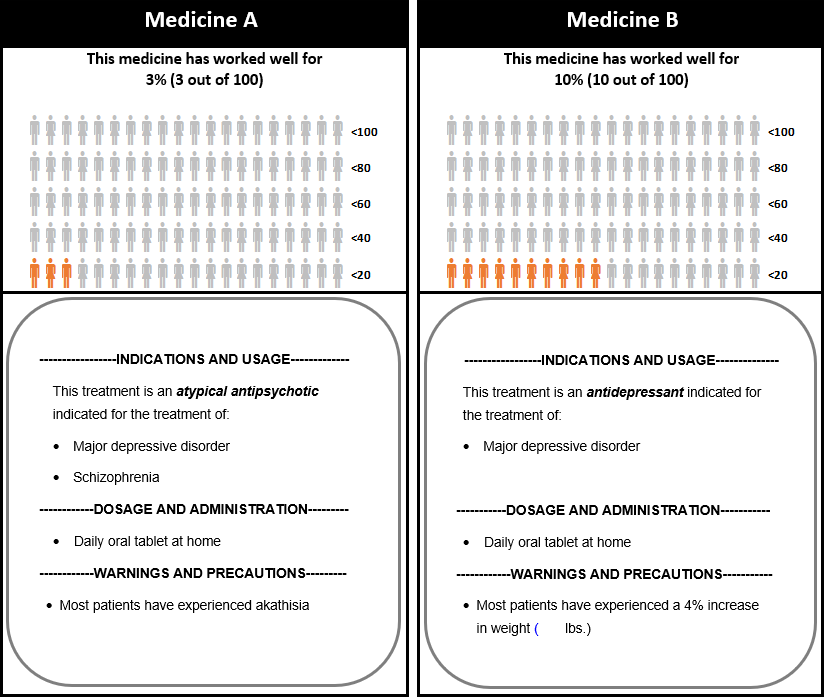


10

Choice1. Which of these medicines would you try **first**?

- - Medicine A
  - Medicine B

**A Few More Questions About You**

Thank you again for your help with this important survey. We are almost finished. We would appreciate your giving us some additional information about you.

B9. What is your gender?

- Female
- Male
- Other or prefer not to answer

B10. What is your marital status?

- Single / never married
- Married / living as married
- Divorced or separated
- Widowed / surviving partner
- Other

B11. Which of the following describes your ethnicity? (*Check only one answer*.)

- Hispanic, Latino or Spanish origin
- Not Hispanic, Latino or Spanish origin [Skip to B13]

B12. Which of the following ethnic groups best describes you? (*Check only one answer*.)

- Mexican, Mexican American or Chicano
- Puerto Rican
- Cuban
- Other Hispanic, Latino or Spanish origin

B13. Which of the following racial groups best describes you? (*Check all that apply*.)

- American Indian or Alaskan Native
- Asian
- African American
- Native Hawaiian or Other Pacific Islander
- White
- Other

B14. How do you get your health insurance? (*Check all that apply*.)

- Through a state or federal insurance exchange
- Through my (or another person’s) employer or union
- Medicare alone
- Medicare and supplemental insurance
- Medicaid
- Veterans affairs
- Other
- I do not have health insurance [Exclusive]

B15. What is the highest level of education you have completed? *(Check only one answer.)*

- Less than high school
- Some high school
- High school or equivalent (such as GED)
- Some college but no degree
- Technical school
- Associate’s degree or 2-year college degree
- 4-year college degree (such as BA, BS)
- Some graduate school but no degree
- Graduate or professional degree (such as MBA, MS, MA, MD, PhD)

B16. Please indicate whether you are currently: (*Check all that apply*.)

- Employed with hourly pay full time
- Employed with salary full time
- Employed with hourly pay part time
- Employed with salary part time
- Self-employed
- Homemaker
- Student
- Retired
- Volunteer work
- Other
- Not working but looking for a job
- Not working and NOT looking for a job
- Unable to work or on disability

**Thank you for participating in this study!**

Your answers to our questions will help doctors and health officials make decisions about new treatments for patients with depression.

Please help us evaluate this survey by selecting what best represents your level of agreement with each of the following statements.

|  | **Strongly disagree** | **Disagree** | **Neither agree nor disagree** | **Agree** | **Strongly agree** |
| --- | --- | --- | --- | --- | --- |
| I ***would*** recommend this survey to friends or loved ones who qualify for this study. | ⭘ | ⭘ | ⭘ | ⭘ | ⭘ |
| I ***would*** trust that other people’s treatment choices in this survey reflect their true preferences. | ⭘ | ⭘ | ⭘ | ⭘ | ⭘ |
| I ***would*** choose the same medicines if I  took this survey again | ⭘ | ⭘ | ⭘ | ⭘ | ⭘ |
| I had enough information to make choices between medicines in this survey. | ⭘ | ⭘ | ⭘ | ⭘ | ⭘ |
| I carefully considered the treatment  options presented in every question. | ⭘ | ⭘ | ⭘ | ⭘ | ⭘ |
