# Supplementary material for: Self-stigmatization and treatment preferences: Measuring the impact of treatment labels on choices for depression medications
Source: PLoS One. 2024 Sep 3;19(9):e0309562. doi: 10.1371/journal.pone.0309562 (PMC11371228; doi:10.1371/journal.pone.0309562)
Supplement: S2 Appendix — (DOCX) [file pone.0309562.s002.docx]

## Data Quality

Data-quality checks included performance on comprehension questions. These questions help identify respondents who did not completely understand the study attributes or question format. Table 1 presents the distribution of responses for the comprehension questions included in the survey.

1. Responses to survey-comprehension questions

|  | **Statistic or Category** | **All Respondents (N = 501)** |
| --- | --- | --- |
| For how many people did the medicine work well?  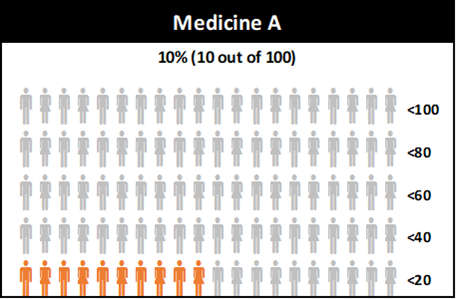 |  |  |
|  | 20 people out of 100 (20%) | 76 (15.2%) |
|  | 90 people out of 100 (90%) | 25 (5.0%) |
|  | 10 people out of 100 (10%) [CORRECT] | 379 (75.6%) |
|  | 5 people out of 100 (5%) | 21 (4.2%) |
| What type of treatment is this medicine? 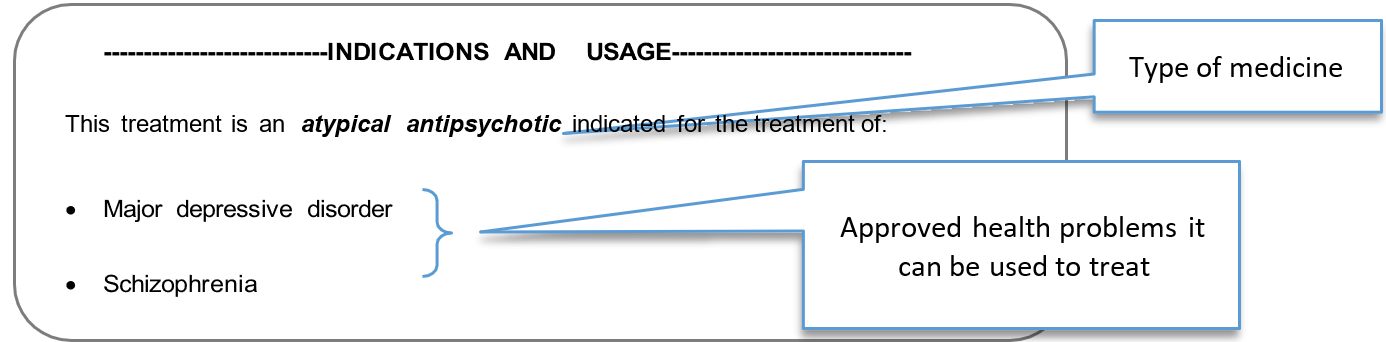 |  |  |
|  | Antidepressant | 78 (15.6%) |
|  | Atypical Antipsychotic [CORRECT] | 378 (75.4%) |
|  | Serotonin-dopamine activity modulator | 15 (3.0%) |
|  | Don't know or not sure | 30 (6.0%) |
| Which of the following health problems is this medicine approved to treat? (Check all that apply.)*  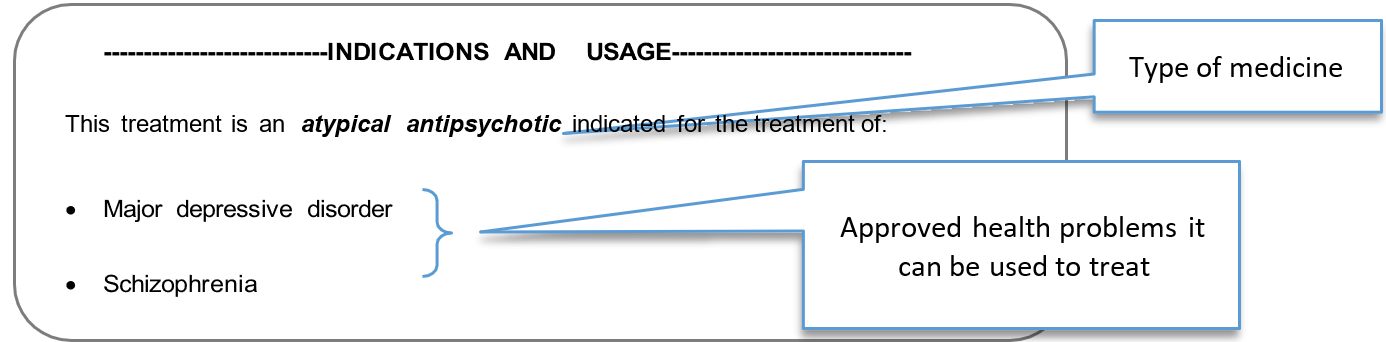 | Major depressive disorder [CORRECT] | 443 (88.4%) |
|  | Anxiety disorder | 65 (13.0%) |
|  | Post-traumatic stress disorder | 30 (6.0%) |
|  | Attention-deficit/hyperactivity disorder | 16 (3.2%) |
|  | Schizophrenia [CORRECT] | 375 (74.9%) |
|  | Bipolar disorder | 27 (5.4%) |
|  | Dementia | 2 (0.4%) |
| Which medicine has worked better to improve people’s mood?  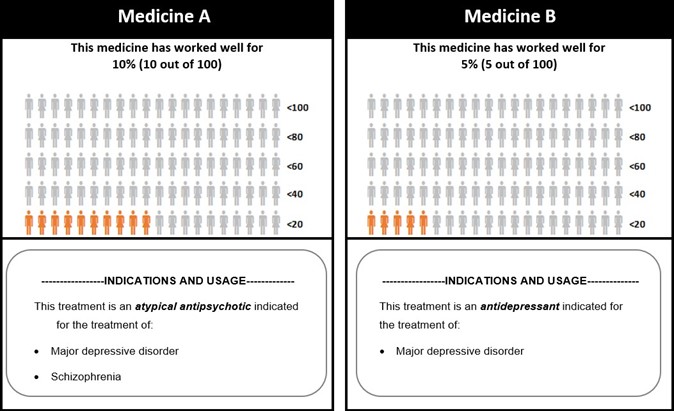 |  |  |
|  | Medicine A [CORRECT ANSWER] | 406 (81.0%) |
|  | Medicine B | 44 (8.8%) |
|  | Both Medicine A and Medicine B have worked about the same | 32 (6.4%) |
|  | Don't know / not sure | 19 (3.8%) |
| Which medicine has been approved to treat more than one kind of health problem? 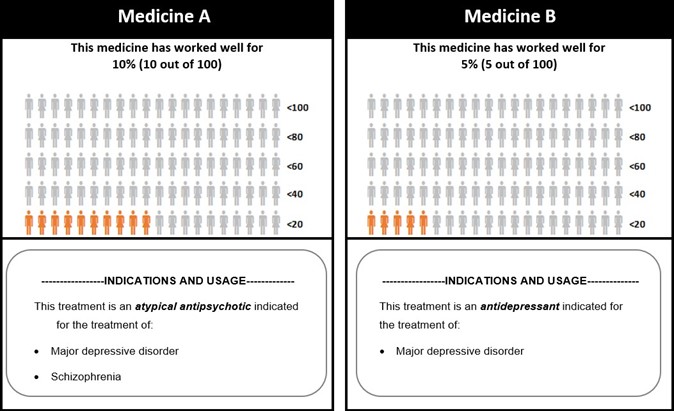 |  |  |
|  | Medicine A [CORRECT] | 398 (79.4%) |
|  | Medicine B | 21 (4.2%) |
|  | Both Medicine A and Medicine B have worked about the same | 54 (10.8%) |
|  | Don't know or not sure | 28 (5.6%) |
| How much weight have people gained within 1 year when they took Medicine A to treat depression symptoms?  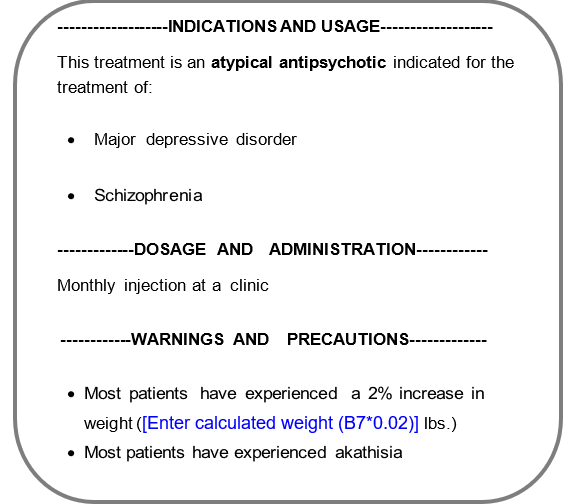 |  |  |
|  | 2% or (weight based on respondent) lbs [CORRECT] | 440 (87.8%) |
|  | 5% or (weight based on respondent) lbs | 20 (4.0%) |
|  | 7% or (weight based on respondent) lbs | 17 (3.4%) |
|  | They did not gain any weight | 2 (0.4%) |
|  | Don't know or not sure | 22 (4.4%) |

On average, respondents provided correct answers to 4.7 (standard deviation=1.7) out of the 6 comprehension questions. Respondents who had more incorrect responses to these comprehension questions, but subsequently saw text explaining why their answer was incorrect, did not have significantly different preferences from respondents with fewer incorrect responses.

The DCE survey was expected to take 20-30 minutes to complete. It included several pages of reading materials and background questions in addition to the discrete-choice questions. On average, respondents took 31 minutes (median 13 minutes) to complete the DCE survey. It is highly unlikely that an attentive respondent could complete the survey in 5 minutes or less. We assessed the distribution of survey completion times and identified respondents who took too little time to complete the survey. We tested the sensitivity of results to inclusion or exclusion of these respondents. Table 2 summarizes the percentiles of the time it took respondents to complete the survey.

1. Time to completion by percentiles (in minutes)

| **Percentiles** | **Minutes** |
| --- | --- |
| 1% | 2 |
| 5% | 6 |
| 10% | 7 |
| 25% | 9 |
| 50% | 13 |
| 75% | 20 |
| 90% | 32 |
| 95% | 51 |
| 99% | 223 |

The treatment alternatives in the choice questions were randomly assigned to the first and second positions and there is no systematic relationship between the alternative placement and the attribute levels shown in each question. Thus, the probability that the actual preferred treatment alternative would appear in the same position for all twelve questions is 0.02%. Only 1.4% of the sample exhibited this straight-lining behavior.

Some respondents selected the alternative with the better level of one attribute in all of the choice questions regardless of other attribute levels. This choice pattern is called attribute dominance. Dominance can indicate the respondent has a particularly strong preference for that attribute, and no combination of levels shown in the other attributes was sufficiently attractive to induce respondents to give up any improvement in the dominant attribute. However, attribute dominance also could be the result of respondents’ simplifying the choice questions to avoid the effort of evaluating tradeoffs.

Table 3 summarizes how many respondents selected the alternative with the better level of each attribute in all choice questions. There was some degree of dominance for 4 of the 5 attributes and the pattern was roughly consistent with the estimated relative attribute importance. We therefore retained these respondents in the analysis since their choices were likely to be a valid expression of preferences.

1. Attribute dominance

| **Attribute** | **Number of people dominating  (N=501)** |
| --- | --- |
| Better level of efficacy | 30 (6.0%) |
| Medication type* | 1 (0.2%) |
| Indication** | 7 (1.4%) |
| Dosage and administration*** | 61 (12.2%) |
| Less weight gain | 37 (7.4%) |
| No akathisia | 62 (12.4%) |

*Always chose atypical antipsychotic. ** Always chose MDD only. *** Always chose daily oral tablet at home.
