# Supplementary material for: Self-stigmatization and treatment preferences: Measuring the impact of treatment labels on choices for depression medications
Source: PLoS One. 2024 Sep 3;19(9):e0309562. doi: 10.1371/journal.pone.0309562 (PMC11371228; doi:10.1371/journal.pone.0309562)
Supplement: Attachment — Submitted filename: Response to reviewers 5-9-24.docx [file pone.0309562.s003.docx]

COMMENT:

Under Introduction:
“it is unknown whether self-stigmatization is exacerbated by association with conditions no experienced by the patients” Recommend reconstructing the sentence for clarity: Like “it remains uncertain whether associations with conditions not directly experienced by the patients exacerbate self-stigmatization.”

RESPONSE:

We have updated the cited text as suggested by the reviewer.

COMMENT:

Under Methods:
“To avoid inducing a reaction from participants in response to these two attributes beyond what would be triggered by the product label” Recommend reconstructing the sentence for clarity: Like “To prevent eliciting a reaction from participants beyond those typically triggered by the product label”
Recommend only using attributes or characteristics and not interchanging them.

RESPONSE:

We have updated the cited text as suggested by the reviewer.

COMMENT:

Define: akathisia and acronyms: SAS

RESPONSE:

We have added the definition for akathisia immediately after it is first mentioned.

COMMENT:

Recommend using voluntarily instead of spontaneously and taking out the extra they in “Importantly, interview participants spontaneously shared concerns about the use of antipsychotics and a treatment indication for schizophrenia as they felt taking such medications would suggest their condition was severe”

RESPONSE:

We have updated the cited text as suggested by the reviewer.

COMMENT:

In “The interviews were conducted individually through videoconferencing and followed a
semi-structured think-aloud protocol” Please describe how a semi-structured think-aloud protocol was followed.

RESPONSE:

We have added additional information explaining the process followed as part of the semi-structured think-aloud protocol. The following text has been added to the methods section.

*During the 1-hour individual interviews we asked participants to read the survey out loud and to comment as needed throughout the document on things that seemed unclear or did not match their own experience with the disease or treatment. In addition, the interviewers probed specifically about the attribute definitions and the understandability of the preference-elicitation exercise.*

COMMENT:

“We accomplished the latter by running a 3-class model, where preferences in class 1 were required to favor treatments without an indication for schizophrenia (i.e., constraining the marginal effect of avoiding schizophrenia to be positive), preferences in class 2 were required to favor treatments with an indication for schizophrenia (i.e., constraining the marginal effect of treating MDD and schizophrenia to be positive). Preference estimates in class 3 were obtained without any parameter constraints.” Combine the sentences with a “while”.

Recommend making this a table for clarity as they are referenced frequently under results. In general, the statistical analysis was difficult to follow and understand. Recommend using more description and rewording sentences.

RESPONSE:

Per the reviewer’s suggestion, we updated the text describing the 3 respondent classes in the analysis and added a new table (Table 2) summarizing the information.

The added text included the following:

*We accomplished the latter by running a 3-class model. Preferences in class 1 were required to favor treatments without an indication for schizophrenia by constraining the marginal effect of having an indication for schizophrenia to be non-positive. Preferences in class 2 were required to favor treatments with an indication for schizophrenia by constraining the marginal effect of treating MDD and schizophrenia to be non-negative. Preference estimates in class 3 were obtained without any parameter constraints. Table 2 summarizes the model specification by class.*

***Table 2. Model specification by class***

| ***Class*** | ***Assumed model specification*** |
| --- | --- |
| *Class 1 – Favors treatments without schizophrenia indication* | *Preference weight for schizophrenia indications was constrained to be non-positive* |
| *Class 2 – Favors treatments with schizophrenia indication* | *Preference weight for schizophrenia indications was constrained to be non-negative* |
| *Class 3 – Unconstrained class* | *All parameters were estimated freely* |

COMMENT:

Under Results:
The first paragraph describing the number of participants and description of encounters can go in under methods.

RESPONSE:

We have moved the cited paragraph to the end of the method’s section.

COMMENT:

Use another phrase for “preference weights” or define what is being referenced by preference weights.

RESPONSE:

We now have defined the results from a logit-based model of preferences as preference weights. This is because the estimates represent the rate of change in the probability of choices with particular attribute levels. The new text in the analysis section reads as follows.

*Estimates from logit-based regression models represent the percentage change in the probability of choice given the attribute levels in the alternatives. For this reason, they also are called preference weights.*

COMMENT:

Reword for clarity: “We also noted a small, marginally statistically significant positive effect of additional context information on the acceptance of atypical antipsychotics and greater chance of indifference about the treatment indications.”

RESPONSE:

We have updated the cited text for clarity. The new text reads as follows:

*We noted that the additional context information changed the acceptance rate of atypical antipsychotics in a small positive way. The context information was also associated with a greater chance of indifference about the treatment indications.*

COMMENT:

Under Discussion:
This sentence is not necessary: “While the effect is relatively small, we found evidence that it is likely pervasive”

RESPONSE:

We would like to maintain this statement as we think it is important to note how widespread the issue of self-stigmatization appears to be in this population based on our results.

COMMENT:

Under “limitations” section: Other limitations identified are including and limited to not addressing other communication strategies, qualitative information on avoidance of adjuvant antipsychotics because of self-stigmatization was not taken from the participants who completed the online survey, and participants answered on the online survey may not reflect in their real-world decision making.

A suggestion to include is giving specific points that doctors can talk about to their patients that subside the patient’s aversion to adjuvant antipsychotics for MDD symptoms. Consider taking about directions for future research.

Limitations accounted for: consistency check was done where only data of good quality was taken into account and statistical lengths were taken to account for different variances in data.

RESPONSE:

In response to the reviewer’s comments we have added the following paragraph to the discussion section.

*In our application, simply highlighting the fact that taking antipsychotics does not require experiencing psychotic episodes reduced avoidance of these treatments. Future research should consider measuring how SDM affect actual patient treatment-taking behavior. This information could help quantify real-world implications of self-stigmatization among MDD patients and support the development of strategies that reduce the avoidance of potentially effective treatments.*
