# Supplementary material for: Self-stigmatization and treatment preferences: Measuring the impact of treatment labels on choices for depression medications
Source: PLoS One. 2024 Sep 3;19(9):e0309562. doi: 10.1371/journal.pone.0309562 (PMC11371228; doi:10.1371/journal.pone.0309562)
Supplement: Attachment — Submitted filename: Response to reviewers 6-20-24.docx [file pone.0309562.s004.docx]

Response to reviewers

| **Comment** | **Response** |
| --- | --- |
| In the study participants were made to read the label and make recommendations. It is important to consider the percentage of patients who read the labels of the medications in detail in real life scenario. | While we presented the information in the form of a product insert, our intention was not to address the impact of the working on a label, but the classification implied by the regulatory approval. In that sense, all we tried to do with the presentation of the information was to mimic a way in which such information may be accessed by patients without unduly highlighting the treatment classification. We have added the following text to clarify this point in the methods section.  *“While we presented the information in the form of a product insert, our intention was not to address the impact of inserts per se, but the information about the product conveyed by regulatory approval in a particular indication or medication category. In that sense, the use of a layout that mimicked the product insert was meant to capture the way such information may be accessed by patients.”* |
| Psychoeducation plays a key role in preventing stigma in most patients with MDD. It is usual practice for psychiatrists to explain the risks, benefits, FDA black box warning, other indications of medications prescribed. Psychiatrists usually explain these to the patient before starting new meds and make them understand why specific medication was chosen for them and how it could help them. These discussions usually help in preventing stigma in most cases. I am curious to know in the study sample if a provider explained them the above and answered all questions. | Our result highlights the importance of the practice highlighted by the reviewer. The information treatment in the experiment—assigned to half of the sample—was meant to capture exactly this. We found that just a little of contextualization of the treatment label was sufficient to address patients’ willingness to forgo treatment efficacy to avoid antipsychotics. We have added the following text (in italics) to the discussion section to address this particular point.  *“Importantly, our results show that psychoeducation can play a key role in preventing stigma in most patients with MDD.* We found evidence that additional information on the use of atypical antipsychotics to treat MDD may increase patients’ willingness to accept a medication classified as an atypical antipsychotic and reduce the impact of an indication for schizophrenia.*”* |
| Study states following: The additional information was intended to provide more context around the use of atypical antipsychotics to treat MDD mimicking the kind of information a patient could receive from physicians to alleviate problems with self-stigmatization. Respondents who were offered the additional information (context arm) were reminded explicitly that patients need not experience psychoses to benefit from antipsychotics. ---- It is important to note: The therapeutic alliance made between patients and psychiatrist usually makes a great difference in stigma, as patients trust on provider is greater to a researcher explaining them. | We agree that, because of the nature of our study, we did not explicitly capture any effects associated with the patient/provider relationship that could moderate the effects we identified in our work. We also agree that future work should work to measure the impact of these relationships on patients’ behaviors and the potential stigma generated by the use of antipsychotics to treat MDD. We have added the following text to the discussion section to address this limitation of our work.  *“The nature of our study, did not explicitly allow us to capture any effects associated with the patient-provider relationship that could moderate the effects we identified in our study. Future studies should measure the impact of these relationships on patients’ behaviors and the potential stigma generated by the use of antipsychotics to treat MDD.”* |
| Also, to some degree, our DCE question design included more information than the limited details given on product inserts to generate meaningful preference weights. ν It is important to know what additional information was added as it changes the validity of the study, and its application to real life scenarios. | We have clarified that the additional information provided was the level of efficacy expected from treatment. This was necessary to evaluate the degree to which respondents were willing to forgo treatment efficacy to avoid specific treatment labeling. We have added the text in italics below to clarify this point.  “Also, to some degree, our DCE question design included more information than the limited details given on product inserts *(i.e., patient-specific treatment efficacy)* to generate meaningful preference weights.” |
| Inclusion criteria: Specification regarding inclusion criteria is missing. Recommended to write clear inclusion criteria. Pl refer to PLOS ONE submission guideline: | Added to the methods section:  “Participants were required to self-report a physician diagnosis of MDD and *must have failed at least one treatment for depression in the past. Answers* to questions about their treatment history were used to verify their report was consistent with diagnosis.” |
| In methods pl include the sample size. | This information is presented at the end of the method’s section per the request of another reviewer.  “A total of 501 respondents were recruited through an online consumer panel between October and November, 2021.” |
| Exclusion criteria needed to be added. | Added to the methods section:  *“Participants who reported having psychotic events were excluded.”* |
| We included two attributes potentially associated with self-stigmatization. First, medication type, which included antidepressants and two different names that could be used to describe the same family of antipsychotics to treat MDD. It is difficult to follow this sentence. Recommend rephrasing | Rephrased to the following:  *“We included two attributes potentially associated with self-stigmatization. First, medication type, which included antidepressants, atypical antipsychotic and serotonin-dopamine activity modulator.”* |
| While the data from this article is informative. Study methodology is unclear with missing information to reproduce the study. Also, study could be more valid if questionnaire used included psychiatrist/ physician involvement in recommending the medication and discussing with patient | Our objective was to evaluate whether self-stigmatization was present among patients with MDD when the treatments they are asked to use are approved for heavily stigmatized mental-health conditions or their naming is unfamiliar or suggestive of use for psychotic episodes. To do this, we asked respondents to make choices between hypothetical treatments that allowed us to identify systematic avoidance of treatment aspects associated with self-stigmatization. We measured the degree to which this avoidance lead to acceptance of reduced treatment efficacy. Our finding suggests that MDD patients indeed seek to avoid medications associated with self-stigmatization at a statistically significant rate. While respondents were not willing to accept large reductions in treatment efficacy to avoid self-stigmatizing features, we found that this behavior was likely pervasive in the population. Additionally, we assessed the impact of adding minimal contextualization of the self-stigmatizing aspects of treatments and found that the additional information was associated with a significant reduction in the avoidance of self-stigmatizing treatment aspects. In that sense, our intention was not to evaluate the right language to discuss treatments with patients, but to evaluate the potential of communication on counteracting self-stigmatization. Our results are consistent with previous work which has found that doctor-patient communication is associated with patient adherence and satisfaction with treatment.  We have added the following text to the discussion to address the reviewer’s concern.  *“Our objective was to evaluate whether self-stigmatization was present among patients with MDD when the treatments they are offered are also approved for stigmatized mental-health conditions. We measured the degree to which this avoidance lead to acceptance of reduced treatment efficacy. Additionally, we assessed the impact of adding minimal contextualization of the self-stigmatizing aspects of treatments. Our intention was not to evaluate the right language to discuss treatments with patients, but to evaluate the potential of communication as a tool to counteract self-stigmatization.”* |
| This article offers valuable insights into self-stigmatization and how it interacts with treatment and medication compliance. Although some of the conclusions were difficult to grasp, overall, the article successfully identifies the impact of self-stigmatization on the treatment of specific mental health issues using a decent sample size. | We hope that the edits to throughout the document in response to the reviewer’s feedback have helped clarified any confusions. |
